# Minimally invasive CT guided treatment of intraspinal synovial cyst

**DOI:** 10.2478/raon-2013-0024

**Published:** 2014-01-22

**Authors:** Sergeja Kozar, Miran Jeromel

**Affiliations:** 1 Clinical Department of Anesthesiology and Intensive therapy, University Medical Centre Ljubljana, Ljubljana, Slovenia; 2 Department of Diagnostic and Interventional Neuroradiology, University Medical Centre Ljubljana, Ljubljana, Slovenia

**Keywords:** intraspinal synovial cyst, polymorbid patient, radiculopathy, pain, percutaneous CT guided treatment

## Abstract

**Background:**

Intraspinal synovial cysts of vertebral facet joints are uncommon cause of radicular pain as well as neurological deficits. They can be managed both conservatively and surgically.

**Case report:**

A 77-year old polymorbid patient presented with bilateral low back pain which worsened during the course of time and did not respond to the conservative treatment. A diagnosis of intraspinal synovial cyst was made using the magnetic resonance imaging (MRI). Percutaneous computed tomography (CT) guided injection with installation of local anesthetic together with corticosteroid and rupture of the cyst was successfully used. A month after the procedure his pain improved, the usage of analgesics diminished and his over-all quality of life improved.

**Conclusions:**

Percutaneous CT guided lumbar synovial cyst treatment is safe and reliable alternative to the surgical treatment in polymorbid patients with radiculopathy who are not able to tolerate general anesthesia and operation.

## Introduction

Radicular pain in lumbar region is a symptom which can have many causes such as herniated nucleus pulposus, migrated disc fragment, lumbar stenosis, facet joint syndrome, malignancy and/or infection.^1^,^2^ Intraspinal synovial cysts are among uncommon causes.^1^

The incidence of lumbar synovial cysts in symptomatic patients is 0.65–2.3% according to a diagnostic method.^2^ Recent reports in literature show that true incidence of the lumbar intraspinal synovial cysts is higher. Higher incidence reflects the improved diagnostic methods.^3^ The disease is more common in older population and in women and is supposed to be related to degenerative changes of the lumbar spine.^1^,^4^

Cysts may be asymptomatic but they usually cause radicular pain, neurogenic claudication, motor deficits, sensory and reflex disturbances or even cauda equina syndrome.^1^ Diagnosis is based on different imaging techniques such as magnetic resonance imaging (MRI), computed tomography (CT) and/or CT myelography.^1^,^5^

## Case report

A 77-year-old patient presented with back pain in the lumbar region and both legs. Lumbar pain started 3 years ago and was primarily described as blunt and propagating along the left lower extremity. Nature of pain changed a year before the presentation into prickling in nature and spontaneous. Symptoms interfered with his daily routine. A similar type of pain affected his right lower limb. According to visual analogue scale (VAS) he evaluated his pain as 8/10. This was improved by bed rest and usage of analgesics and described as VAS 4/10.

The patient had a complete occlusion of left internal carotid artery, both vertebral arteries and 70% stenosis of right internal carotid artery. He had already suffered two myocardial infarctions as well as an ischemic stroke. His other comorbidities included chronic atrial fibrillation, congestive heart failure, peripheral arterial occlusive disease, arterial hypertension, hyperlipidemia, hypercholesterolemia, benign prostatic hyperplasia and chronic renal insufficiency.

**FIGURE 1.**
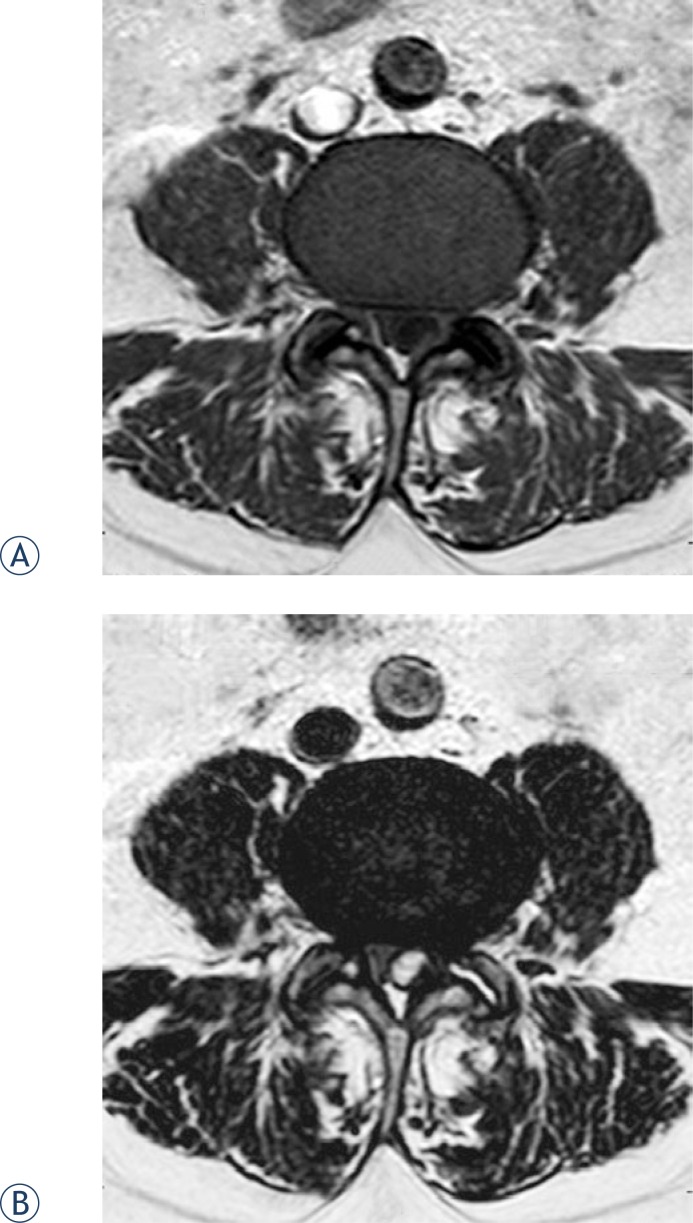
Baseline (preoprocedural) MRI in an axial plane at the level of the L4–L5 lumbar segment. Hypointense well delineated cystic formation compressing dural sac from the left dorsolateral side is seen on T1 SE image (A). The cyst that is hyperintense on T2 FSE image shows continuation with the left facet joint (some fluid can also be seen in the degenerated facet joint) (B).

MRI revealed well delineated intraspinal cystic formation compressing dural sac from the left dorsolateral side at the level of fourth and fifth lumbar facet joint. The dural sac was significantly compressed and displaced by the cyst filling more than half of the normal canal. The communication of the cyst with the left facet joint was seen. Presence of the fluid within the degenerated facet joint was additional finding that supported the diagnosis of intraspinal synovial cyst (Figure 1).

Due to complex medical history the decision for non-surgical, percutaneous CT -guided intervention was made. Standard monitoring (electrocardiogram, non-invasive blood pressure measurement and pulse oximetry) was used. An intravenous line was inserted. The patient received cefazoline 2 g and paracetamol 1 g 30 minutes before the start of the procedure and was placed in the prone position on the CT scanner’s table. The procedure was performed under strict aseptic conditions by using CT scanner with 82 cm gantry opening (Sensation Open, Siemens Healthcare, Erlangen, Germany). The scanner with STRATON X-ray *tube* technology *enables an* acquisition of *40 slices. T*he *wide gantry opening* ensures an easy patient access. The following CT protocol was used for CT guidance: low dose scans (120 kV, 112 mAs) with rapid 360^0^ gantry rotation speed (1 second) and 0.6 mm section collimation (slice thickness). A puncture point (overlying the left L4–L5 facet joint) was selected on axial CT scan. Local infiltration with 2% lydocaine 2 ml was used.

**FIGURE 2.**
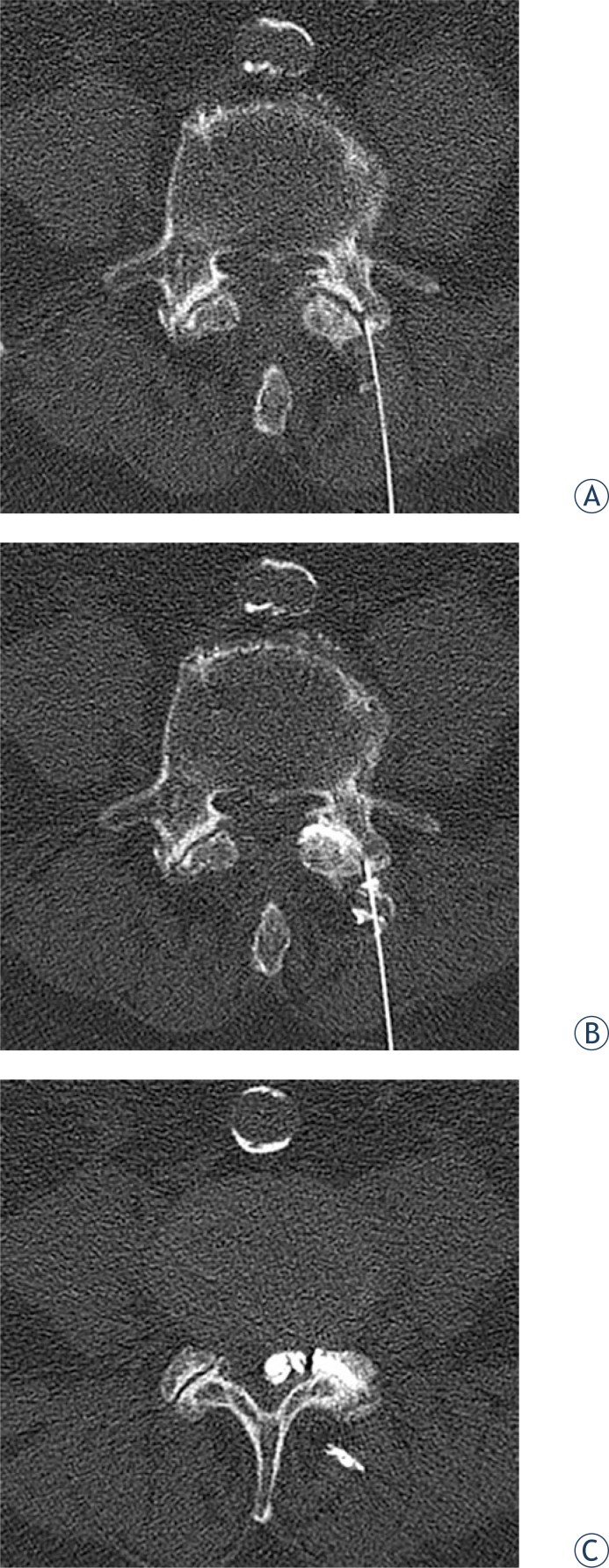
Intraprocedural CT in an axial plane at the level of the L4–L5 lumbar segment. A 22G needle that has entered a posterior part of a target facet joint can be seen (A). Iodine contrast media instilled through the needle has filled the facet joint, confirming the needle tip position inside the joint (B). Continuous contrast instillation resulted in intraspinal contrast opacification of the cyst (confirming the diagnosis of intraspinal synovial cyst) (C). A loss of resistance was felt at the time of cyst rupture. The procedure was terminated at that point.

A 22 gauge (G) atraumatic spinal needle (RapID Portex, Smiths Medical, London, United Kingdom) was advanced forward targeting the joint. The position of the needle was confirmed with subsequent CT scans. After the insertion of the needle into the facet joint space, iodine contrast media iohexol (Omnipaque 300 mg/mL, GE Healthcare, Amersham, United Kongdom) 10 ml was instilled. A communication between the joint space and the intraspinal cyst was proved, confirming the diagnosis of the synovial cyst. A mixture of local anaesthetic 1% lydocaine 1 ml and methylprednisolone 125 mg was injected into the lumen of the cyst. Additional sterile saline was installed in order to distend and rupture the cyst. The patient described pain in the spinal region and both legs during this part of the procedure, which partially responded to intravenous 0.05 mg of alfentanyl application. The loss of resistance suggested the rupture of the cyst and this was confirmed by CT scan (Figure 2). At the moment of the rupture the pain disappeared. The procedure was terminated by this event. The patient was haemodinamically stable during the procedure.

Immediately following the procedure a sterile dressing was placed over the skin entry. The patient was observed and monitored in recovery unit for two hours. A strict body rest was maintained during this period of time. The patient did not require any additional analgesics. He was discharged next day. Instructions about the wound care and observation were given to him.

A short-term follow-up with the referring physician as well as with the patient himself revealed the partial relief of radicular symptoms a month after the procedure. His pain still persists but is described as blunt and without propagation. Prickling pain in both his lower extremities disappeared. His only pain therapy is weak opioid before sleep.

## Discussion

As already mentioned, intraspinal synovial cysts are uncommon cause of radicular pain and are located on the internal and posterolateral side of the spinal canal.^1^ Intraspinal synovial cysts are continuous with the facet joints and lined with epithelial lining. They are typically located posterior to the thecal sac; this is thought to be related to the presence of ligamentum flavum which represents a barrier to anterior cyst formation.^3^ Majority of these cysts occur in the segments of lumbar spine with the predilection for the fourth and fifth lumbar vertebra as described in our patient.^1^,^4^–^6^ Cysts in the cervical spine are uncommon and those in the thoracic spine are even more rare.^1^

Pathogenesis of synovial cysts is not well understood. A chronic degenerative process causes the protrusion of the synovial membrane through the defects of the joint capsule. Consequently formation of the cavity filled with synovial fluid occurs.^1^,^2^,^5^ Therefore, the pathognomic imaging finding is a direct communication between the cyst and the facet joint.^3^,^5^ This was clearly demonstrated in our case as well. Imaging with iodine contrast media proved the communication between the cyst and facet joint and, thereby, confirmed the diagnosis. This theory is also supported by the fact that most of the patients with synovial cysts suffer from facet joint osteoarthritis and disk degeneration.^2^ Since the cysts arise at the most unstable segments of the spine, instability is a pivotal factor. Another theory implicates trauma to the spine.^1^ Our patient had no history of previous spinal trauma. Diagnostic imaging of lumbosacral region revealed degenerative changes.

The clinical presentation of the intraspinal synovial cysts depends on the size, site and relationship to the adjacent structures. Cysts may be asymptomatic but they usually cause radicular pain, neurogenic claudication, motor deficits, sensory and reflex disturbances or even cauda equina syndrome.^1^ The most common presentation is a radicular pain.^7^ All these presentations develop gradually; the acute onset of symptoms may be associated with a sudden cyst distension and/or compression of parts of nervous system, usually caused by intercystic haemorrage.^1^ Our patient suffered from bilateral low back pain. He also experienced weakness in both lower extremities, which besides the pain interfered with his general activities and ability to walk. All symptoms progressed during the course of time. No other neurological deficits were described by the patient or clinicians.

The diagnosis of intraspinal synovial cysts is based on MRI, CT or CT myelography.^1^,^5^,^8^ The latter is used when MRI is not available or cannot be performed.^1^ A typical synovial cyst is defined as round or ovoid collection arising from the facet joint. It is composed of varying types of fluid.^3^ According to MRI evaluation in our case, the cyst contained a fluid with a density similar to clear fluid (the signal was similar to cerebrospinal fluid). However, due to the viscosity of the fluid, the aspiration with 22 G needle was not possible.

Intraspinal synovial cysts can resolve spontaneously, but they usually require the treatment. The treatment (besides surgical) includes bed rest, analgesics, physical therapy, transcutaneous electrical stimulation (TENS), as well as minimal invasive methods such as epidural/intraarticular corticosteroid injections with or without rupture of the cyst and CT or endoscopy guided aspiration of the cyst.^1^,^2^ The conservative treatment was used in our case, but the symptoms worsened during the course of time. Therefore, the interventional treatment became an option.

Non-surgical interventions include installation of corticosteroids and/or local anesthetics. The aim is to prevent the recurrence of the cyst and inflammation.^3^

The rupture of the cyst can be achieved with the additional injection of normal saline. An immediate complete relief is described by the majority of patients. According to reports of some authors, significant long term effect cannot be achieved.^3^,^7^ A retrospective study conducted by Martha *et al.* confirmed a sufficient symptomatic improvement in 46% of treated patients as well as avoidance of the surgical treatment. A cyst rupture was described in all of them. Patients, who underwent surgical procedure after the minimal invasive procedure, reported the significant improvement in disability, back and leg pain.^9^ Another retrospective study proved pain relief to be associated with the rupture of the cyst.^6^ In case of the severe degenerated facet joint with a very narrow joint space, the posterior facet puncture could be challenging. An alternative approach can be used in such cases. The cyst can be punctured directly through vertebral lamina. The later approach is commonly used in ganglion cysts that have no connections with facet joints.^1^

The advantage of CT over fluoroscopy is direct, reliable puncture of the cyst without dural violation and was a method of choice in our case as well. Another advantage of the procedure is that it can be repeated if necessary. Common risks include infection, bleeding or damage to nervous structures.^3^,^7^ None of the complications occurred in our case; the patient received antibiotic prophylaxis and the procedure was performed under strict aseptic conditions. His therapy with warfarine was stopped and changed into low molecular weight heparine to minimize the possibility of bleeding.

Surgical treatment includes different procedures (laminectomy, facetectomy, flavectomy together with cyst excision and microsurgical procedures). The aim of these procedures is a complete resection of the cyst as well as the treatment of concomitant disease.^1^,^2^,^10^ The surgical treatment is the most effective treatment option with very rare instances of recurrence of the cyst.^3^,^11^

However, the optimal treatment strategy of synovial cysts remains controversial.^1^,^4^ The surgical treatment can be recommended when conservative methods (bed rest, analgesics, physiotherapy) fail to alleviate symptoms or as soon as neurological deficits appear. Older and high risk patients may also benefit from percutaneous interventions.^1^–^3^,^10^,^11^ Our patient was not able to tolerate the surgical procedure due to all his comorbidities, especially his cardiac and vascular disease.

## Conclusions

Percutaneous CT- guided lumbar synovial cyst treatment is safe and reliable alternative to the surgical treatment in polymorbid patients with radiculopathy unable to tolerate general anesthesia and operation.
